# Fungal scleritis masquerading as surgically induced necrotizing scleritis: a case report

**DOI:** 10.1186/1752-1947-7-288

**Published:** 2013-12-30

**Authors:** Srikant Kumar Sahu, Sujata Das, Debabrata Sahani, Savitri Sharma

**Affiliations:** 1 Cornea and Anterior Segment Service L V Prasad Eye Institute, Bhubaneswar, Orissa 751 024, India; 2 Maa Tarini Netradham, Keonjhar, Orissa, India; 3 Ocular Microbiology Service L V Prasad Eye Institute, Bhubaneswar, Orissa 751 024, India

**Keywords:** Fungal scleritis, Surgically induced necrotizing scleritis

## Abstract

**Introduction:**

The object of this case is to report the clinical findings, microbiological findings and management of a case of fungal scleritis following cataract surgery, which mimicked surgically induced necrotizing scleritis.

**Case presentation:**

A 72-year-old Asian (Indian) man presented with scleritis following cataract surgery at another facility. He had been treated elsewhere for suspected scleritis, primarily with steroids followed by empiric antibiotic and antifungal agents. At our institute he underwent a complete microbiological workup and a scleral patch graft. The scleral scraping revealed fungal filaments. He was treated postoperatively with topical and systemic antifungal agent along with topical cyclosporine. The follow-up examination at 5 months revealed that the scleral patch graft was successful in maintaining the integrity of his globe and restoring partial vision.

**Conclusions:**

Fungal scleritis may mimic surgically induced necrotizing scleritis. Early diagnosis and prompt management can prevent progression of the disease and further devastating complications.

## Introduction

Surgically induced necrotizing scleritis (SINS), a rare complication of cataract surgery, is described as inflammation and necrosis of the sclera adjacent to the site of surgery [1]. It has been reported to occur after cataract, retinal detachment, keratoplasty, trabeculectomy, and strabismus surgery [1]. Typically scleral melt develops adjacent to the wound. It has a variable latent period. SINS is usually a non-infective necrotizing disease and the treatment includes topical and systemic corticosteroids [1].

Fungal sclerokeratitis has been described as a rare postoperative complication of cataract surgery [2]. It usually involves the scleral tunnel and extends to the cornea. Steroids are contraindicated in the management of fungal infection [3].

We report a rare case of fungal scleritis following cataract surgery that mimicked SINS.

## Case presentation

A 72-year-old Asian (Indian) man presented to our Cornea and Anterior Segment Service with a referral diagnosis of scleritis in his left eye. He had a history of having had cataract surgery in his left eye at another facility 6 weeks earlier with good postoperative vision.

The medical history suggested that his postoperative vision was good until day five. He complained of severe pain and diminution of vision in his operated left eye on the fifth postoperative day. With a working diagnosis of SINS his primary ophthalmologist put him on systemic and topical corticosteroids. However, when his vision continued to decrease and pain persisted, his ophthalmologist started topical natamycin every hour, and topical ciprofloxacin every 2 hours while continuing topical prednisolone acetate every 2 hours. This new treatment, however, did not help improve his condition.

**Figure 1:**
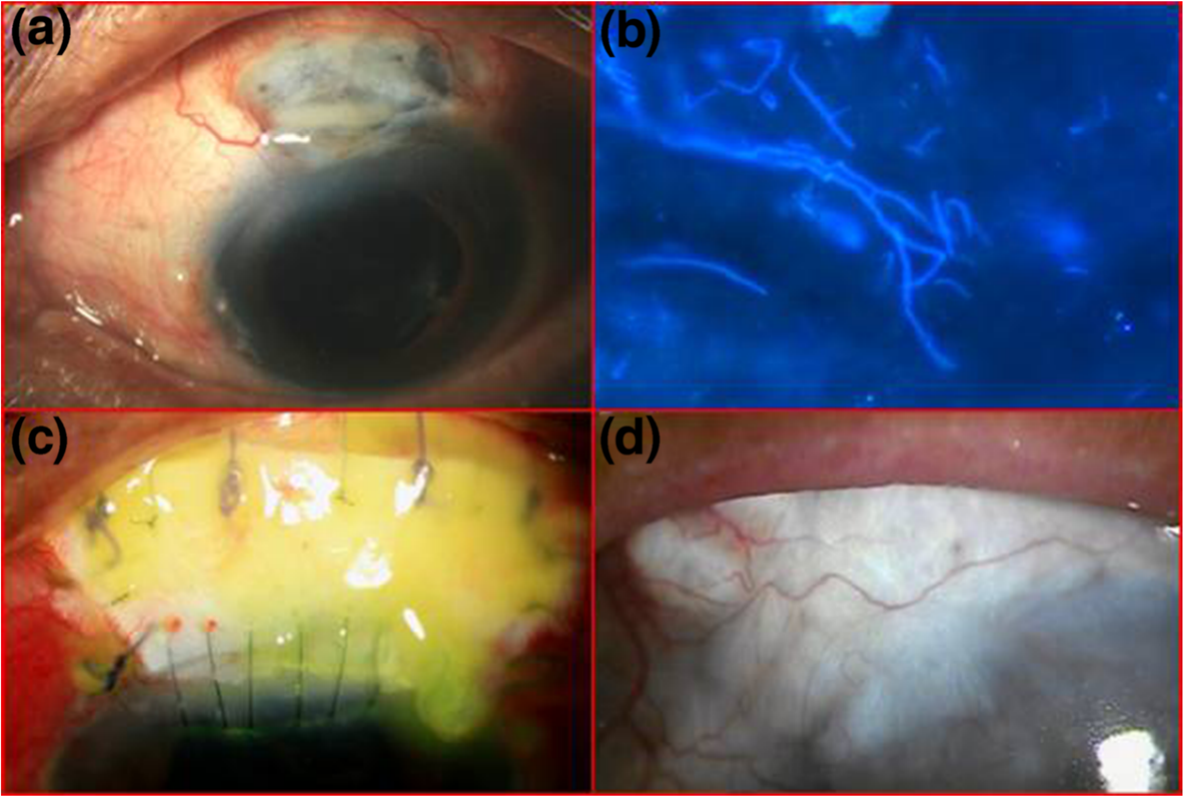
(a) Diffuse slit-lamp view showing the area of necrosis along with the exudate on it; (b) fungal filament seen in potassium hydroxide and calcofluor white stain (×400); (c) postoperative day 1 showing the patch graft; (d) vascularization over the graft after 5 months.

At presentation to us his visual acuity was counting fingers at 1 meter in his right eye and 2.5 meters in his left eye. Apart from a cataract (grade 3 nuclear sclerosis) his right eye was apparently within normal limits. A slit-lamp examination of his left eye showed a well demarcated area of scleral necrosis predominately involving the area posterior to the posterior lip of the scleral incision. The anterior lip of the scleral wound was also involved (Figure 1a). The sclera around the necrotic region was congested. His cornea was clear. There were 2+ cells in the anterior chamber. As he was very symptomatic a detailed fundus evaluation could not be done but his posterior pole appeared to be within normal limits.

A presumptive diagnosis of SINS was made. The wound was scraped, and necrotic tissue was debrided and sent for microbiological evaluation. Microscopy in potassium hydroxide and calcofluor white stain (Figure 1b) revealed fungal filaments. Donor sclera of an appropriate size (7.5mm × 11mm) was patched over the thinned sclera (Figure 1c). The donor sclera which was preserved in absolute alcohol was obtained from the eye bank. After cleaning the preserved sclera with Ringer’s lactate solution and 5% povidone solution, it was cut according to the area excised. It was sutured with 6–0 polyglactin suture with the surrounding sclera and with 10–0 nylon suture with the limbal portion of cornea.

He was started on topical natamycin (5%) every hour, cyclosporine (0.1%) two times a day, homatropine three times a day and systemic itraconazole 100mg two times a day. A systemic evaluation was done postoperatively to rule out any other etiology of the scleritis. Blood studies showed a normal total and differential count and an erythrocyte sedimentation rate of 14mm/hour. Rheumatoid factor and anti-nuclear antibody were negative. Liver function tests were within normal limits. All antifungals were discontinued on the 18th day following the scleral graft as there was no evidence of active infection. He was initiated on topical prednisolone acetate (1%) eight times a day and homatropine (2%) eye drops at bed time. He was regularly examined by the primary ophthalmologist. At the last examination in the fifth postoperative month, he was symptom free and his vision was 6/36. An area of retinal pigment epithelium alteration was seen hazily due to posterior capsular opacity. The graft was healthy and vascularized (Figure 1d).

## Discussion

An infective complication after cataract surgery is a serious threat to vision. There are few published data on corneoscleral wound infections. A poorly constructed wound, loose or broken sutures, and associated dacryocystitis have been identified as important predisposing factors for wound infection in these reports [4-6]. The proximity of the outer lip to the conjunctival flora may actually increase the chance of inoculation of the wound.

Garg *et al.* have reported seven cases of fungal infection of sutureless self-sealing incisions. One of the seven patients presented with scleritis, but there was no scleral melt [2]. Moriarty *et al.* have reported four cases of fungal corneoscleritis following radionecrosis. All of them presented after a long duration, 13 to 20 years after radiotherapy. Three patients had calcific plaques which were thought to be the nidus for infection. They suggested adequate debridement of necrotic tissue and antifungal therapy before the graft is performed [7].

The major differential diagnosis of scleral necrosis in the postoperative period is SINS. O’Donoghue *et al.* have described the factors precipitating SINS and the response to treatment. They also reported that corticosteroids and immunosuppressives are the mainstay of therapy [1].

Our patient presented with a focal area of scleral inflammation, necrosis, and melt occurring adjacent to the site of the previous scleral incision as is seen in SINS [1]. Fungal scleritis might have been the primary event in our patient because the disease stopped progressing after surgical removal of the exudates and necrotic tissue, and discontinuation of corticosteroids. The topical and systemic corticosteroids used in the initial phases by the primary physician could have been the cause of rapid progression.

Management of such cases needs a microbiological workup. Debridement of necrotic tissue helps reduce the load of the organisms. A scleral patch graft has proven to be an effective method of closing the scleral defect [8]. Although the scleral patch graft was successful in our case in restoring functional vision, in infective conditions it should only be considered to preserve the integrity of the globe. Prior medical management and close follow up will prevent recurrence.

Topical cyclosporine has been used in the management of therapeutic keratoplasty for a mycotic keratitis [9]. It allows the surgeon to decrease or avoid the use of topical corticosteroid in the management of therapeutic keratoplasty for mycotic keratitis. It modulates the local immune response by suppressing antigen-activated T lymphocytes while preserving the immune system’s antimicrobial action. Furthermore cyclosporine A has also been shown to have a statistically significant suppressive effect on fungal growth [10,11]. To reduce the amount of inflammation associated with scleral graft, cyclosporine may be used where steroids cannot be used as in our case.

The presence of a systemic disorder is quite common in postoperative scleritis. O'Donoghue *et al*. reported the presence of an underlying medical disorder of which the most common was connective tissue disorder in 63% of patients diagnosed to have postoperative scleral necrosis [1]. Our patient had none; search for these systemic conditions and appropriate treatment is essential.

To treat a patient for SINS a complete microbiological systemic workup and, if necessary, surgical management of the defect can prevent devastating complications.

## Conclusions

Fungal scleritis may mimic SINS. A scraping from the base aids in the diagnosis and appropriate management of the patients. A patch graft is an option in the management of cases where necrosis of sclera tissue threatens the integrity of the globe. If there is no evidence of residual or recurrence then steroids can be started to maintain the graft.

## Consent

Written informed consent was obtained from the patient for publication of this case report and accompanying images. A copy of the written consent is available for review by the Editor-in-chief of this journal.

## Competing interests

The authors declare that they have no competing interests.

## Authors’ contributions

SKS; clinical management, surgeon, drafting of manuscript. SD; correction of manuscript and clinical management of the case. DS; primary physician, postoperative management, and correction of the manuscript. SS; a microbiologist who aided in the management of the case. All authors read and approved the final manuscript.
